# Gla-rich protein function as an anti-inflammatory agent in monocytes/macrophages: Implications for calcification-related chronic inflammatory diseases

**DOI:** 10.1371/journal.pone.0177829

**Published:** 2017-05-18

**Authors:** Carla S. B. Viegas, Rúben M. Costa, Lúcia Santos, Paula A. Videira, Zélia Silva, Nuna Araújo, Anjos L. Macedo, António P. Matos, Cees Vermeer, Dina C. Simes

**Affiliations:** 1 Centre of Marine Sciences (CCMAR), University of Algarve, Faro, Portugal; 2 GenoGla Diagnostics, Centre of Marine Sciences (CCMAR), University of Algarve, Faro, Portugal; 3 UCIBIO@REQUIMTE Departamento Ciências da Vida, Faculdade de Ciências e Tecnologia, Universidade NOVA de Lisboa, Caparica, Portugal; 4 UCIBIO@REQUIMTE, Departamento de Química, Faculdade de Ciências e Tecnologia, Universidade Nova de Lisboa, Caparica, Portugal; 5 Centro de Investigação Interdisciplinar Egas Moniz, Egas Moniz-Cooperativa de Ensino Superior CRL, Caparica, Portugal; 6 VitaK, Maastricht University, Maastricht, The Netherlands; Institut d'Investigacions Biomediques de Barcelona, SPAIN

## Abstract

Calcification-related chronic inflammatory diseases are multifactorial pathological processes, involving a complex interplay between inflammation and calcification events in a positive feed-back loop driving disease progression. Gla-rich protein (GRP) is a vitamin K dependent protein (VKDP) shown to function as a calcification inhibitor in cardiovascular and articular tissues, and proposed as an anti-inflammatory agent in chondrocytes and synoviocytes, acting as a new crosstalk factor between these two interconnected events in osteoarthritis. However, a possible function of GRP in the immune system has never been studied. Here we focused our investigation in the involvement of GRP in the cell inflammatory response mechanisms, using a combination of freshly isolated human leucocytes and undifferentiated/differentiated THP-1 cell line. Our results demonstrate that VKDPs such as GRP and matrix gla protein (MGP) are synthesized and γ-carboxylated in the majority of human immune system cells either involved in innate or adaptive immune responses. Stimulation of THP-1 monocytes/macrophages with LPS or hydroxyapatite (HA) up-regulated GRP expression, and treatments with GRP or GRP-coated basic calcium phosphate crystals resulted in the down-regulation of mediators of inflammation and inflammatory cytokines, independently of the protein γ-carboxylation status. Moreover, overexpression of GRP in THP-1 cells rescued the inflammation induced by LPS and HA, by down-regulation of the proinflammatory cytokines TNFα, IL-1β and NFkB. Interestingly, GRP was detected at protein and mRNA levels in extracellular vesicles released by macrophages, which may act as vehicles for extracellular trafficking and release. Our data indicate GRP as an endogenous mediator of inflammatory responses acting as an anti-inflammatory agent in monocytes/macrophages. We propose that in a context of chronic inflammation and calcification-related pathologies, GRP might act as a novel molecular mediator linking inflammation and calcification events, with potential therapeutic application.

## Introduction

Inflammation and calcification are common events in chronic inflammatory diseases, such as atherosclerosis and osteoarthritis, involving infiltration of monocytes and accumulation of macrophages [1–5]. The interplay between inflammatory and pathological calcification processes is currently widely accepted, and macrophages are known key players signaling extracellular matrix (ECM) degradation, resident tissue cells differentiation and calcification [2, 6]. Although many aspects concerning the molecular mechanisms involved in pathological calcification remain to be elucidated, features such as chronic inflammation, increased extracellular matrix (ECM) remodeling, loss of anticalcific mechanisms leading to proliferation and differentiation of resident cells, and the release of calcifying extracellular vesicles (EVs) are known features contributing to the development of calcific lesions [7–11]. In atherosclerosis, early plaque calcification associates with macrophage accumulation, and macrophage infiltration and inflammation have been shown to precede osteogenic conversion of vascular smooth muscle cells (VSMCs) and the release of EVs [12, 13]. In osteoarthritis, synovium inflammatory and destructive responses, leading to increased cartilage degradation and calcification, are largely promoted by activated synovial macrophages [14–16]. Activated macrophages at sites of tissue damage produce high levels of matrix metalloproteinases, cysteine endoproteases, cytokines, and catabolic prostaglandins which will enhance elastin and collagen degradation leading to remodeling and structural changes of the ECM, promoting calcification [12, 17, 18]. Moreover, macrophages have been shown to regulate vascular calcification through the release of osteogenic factors capable of inducing VSMCs osteochondrogenic differentiation [2, 6]. It has been recently proposed that macrophages release calcifying EVs loaded with mineralization related factors, capable of accelerating ECM calcification [13]. In turn, increased matrix degradation and calcification promote macrophage pro-inflammatory responses. Basic calcium phosphate (BCP) crystals, which are the major forms of mineral crystals associated with both atherosclerosis and osteoarthritis, can activate macrophages inducing proinflammatory responses with increased tumor necrosis factor alpha (TNFα), interleukin-(IL) -1β and IL-8 cytokine production [19]. Also, certain BCP-coated crystals, such as fetuin-A-containing calciprotein particles (CPP), decrease macrophage cytokine production when compared to naked crystals [20]. It is suggested that pathological calcification is not solely a passive consequence of chronic inflammatory disease but is also involved in a positive feed-back loop of calcification and inflammation, driving disease progression [2, 12, 14, 16]. Nevertheless, additional knowledge is required to understand the crosstalk between the events leading to a calcification/inflammation pathological feeding cycle. The identification of molecules involved in both processes would represent a potential therapeutic value targeting calcification-related chronic inflammatory diseases. Vitamin K, known to be critical for preventing soft tissue mineralization has been demonstrated to be able to induce anti-inflammatory responses in macrophages playing a protective role against inflammation [21–23]. Vitamin K is an essential cofactor for the post-translational modification of vitamin K-dependent proteins (VKDPs), required for the modification of specific glutamic acid (Glu) residues to calcium binding γ-carboxyglutamic acid (Gla) residues, in a reaction catalysed by the γ-glutamyl carboxylase (GGCX) enzyme [24, 25]. Continuous recycling of the active form of vitamin K is performed by vitamin K epoxide reductase (VKOR), which is inhibited by warfarin leading to impaired γ-carboxylation of VKDPs (as described in detail previously [26]). Additional functions of vitamin K, such as anti-inflammatory, inhibitor of tumor progression, as well as transcriptional regulator of osteoblastic genes, have been recently proposed as a direct effect of vitamin K, described to be independent of warfarin and formation of Gla residues [27]. However, the association of VKDPs to these new vitamin K functions, such as inflammation, has been poorly explored until now. Further knowledge on the role of VKDPs in inflammatory processes should clarify the possible association of vitamin K in inflammation related to γ-carboxylation.

Gla-rich protein (GRP) is the newest member of the VKDP family recently shown to be involved in the crosstalk between inflammation and calcification of articular tissues in osteoarthritis [16]. Nevertheless, the involvement and relevance of GRP and its γ-carboxylation status in the immune cells inflammatory responses has never been studied. We and others have proposed GRP as a negative regulator of osteogenic differentiation [28], a modulator of calcium availability in the ECM [29, 30], and an inhibitor of calcification in the cardiovascular [31] and articular systems [16]. γ-carboxylation of GRP was demonstrated to be essential for its calcification inhibitory function [16, 31], similarly to matrix Gla protein (MGP) that is widely accepted to play a pivotal role in preventing soft tissue calcification and local mineralization of the vascular wall and articular cartilage [25, 32]. Our previous studies also shown that down-regulation of mediators of inflammation in chondrocytes and synoviocytes treated with GRP is apparently independent of its γ-carboxylation status [16]. GRP calcium binding properties [29, 33] and association to calcification processes [16, 30, 31, 34] indicate that its function might be related with prevention of calcium-induced signaling pathways and to inhibition of crystal formation/maturation by direct mineral-binding. Moreover, GRP is also involved in the mineralization-competence of VSMCs-derived EVs possibly associated with the fetuin-A-MGP calcification inhibitory system [31]. In this work, we aimed to study the capacity of cells of the human immune system for the synthesis and carboxylation of GRP, and further highlight the association of GRP with inflammatory responses of THP-1 monocyte/macrophage cells. Our results indicate GRP as an anti-inflammatory agent in monocytes/macrophages, and we suggest GRP as a new molecular link between inflammation and calcification, with important implications on the development and progression of calcification-related chronic inflammatory diseases.

## Materials and methods

### Ethics statement

Buffy coats from adult healthy volunteers were obtained at the national blood bank (IPST, Instituto Português do Sangue e da Transplantação, Lisboa), after written informed consent of all participants and approval by the “Conselho Directivo” of IPST. All principles of the Declaration of Helsinki of 1975, as revised in 2000, were followed.

### Isolation of human leukocytes

Peripheral blood mononuclear cells (PBMCs) and polymorphonuclear cells (PMNs) were isolated using a Ficoll (Biochrom AG, Germany) gradient centrifugation, and contaminating erythrocytes present in the PMN pellet were lysed with an ammonium chloride solution (0.8% (w/v) NH_4_Cl, 0.1 mM EDTA, buffered with KHCO_3_ to pH 7.2–7.6) in a ratio 1:9 (v/v) cell suspension/solution. Monocytes and T lymphocytes were independently isolated from the PBMC fraction, by positive selection using MACS magnetic beads (Miltenyi Biotec, Germany) against CD14 and CD3 respectively, following manufacturer’s instructions. Neutrophils were isolated from the PMN fraction by negative selection, using EasySep Human Neutrophil Enrichment Kit (StemCell Technologies, France) according to manufacturer’s instructions. Each leukocyte subset was obtained from 3 different donors (n = 3). The non-circulating leukocytes, monocyte-derived macrophages and dendritic cells, were obtained by culturing monocytes according to established protocols [35].

### Flow cytometry

To confirm cell purity, isolated leukocyte subsets were analyzed by flow cytometry with an Attune® Acoustic Focusing Cytometer (Applied Biosystems) by accessing their dispersion pattern or after staining with specific cell surface markers, such as anti-human CD3 antibody (BioLegend, San Diego, CA, USA) to stain T lymphocytes, and anti-human CD16 antibody (BD Biosciences, San Jose, CA, USA) to stain neutrophils.

### Cell culture

Freshly purified monocytes were cultured in complete RPMI 1640 media (1% L-glutamine (R&D Systems, Minneapolis, MN, USA), 10% heat-inactivated FBS, 1% penicillin-streptomycin (PS), 1% non-essential amino acids and 1 mM sodium pyruvate (all from Sigma-Aldrich, St. Louis, MO, USA)). For monocyte-derived cultures, cells were plated in 24 wells plate (1×10^6^ cells per well) in complete RPMI 1640 medium with 75 U/ml IL-4 and 1000 U/ml recombinant human granulocyte macrophage colony-stimulating factor (GM-CSF) (both from R&D Systems) for 6 days, to obtain monocyte-derived dendritic cells (MoDC), or with 1000U/ml GM-CSF (Peprotech, London, UK) for 7 days, to obtain monocyte-derived macrophages. Cultures were incubated at 37°C in a 5% CO_2_ humidified atmosphere and fresh medium was added every 2/3 days. THP-1 cell line was kindly given by Dr. Nuno Santos (CBME, University of Algarve, Faro, Portugal) and was cultured according to ATCC instructions, in RPMI 1640 with L-Glutamine (Lonza, Visp, Switzerland), 10% heat-inactivated FBS (Invitrogen, Carlsbad, CA, USA) and 1% PS (Thermo Scientific, Waltham, MA, USA). Differentiation into macrophagic THP-1 (THP-1 MoM) was achieved by culturing cells in 25 ng/ml of phorbol 12-myristate 13-acetate (PMA) (Sigma-Aldrich) in complete RPMI for 48h.

### Inflammatory stimulation assays

THP-1 and/or THP-1 MoM were seeded in 12-well plates at 1x10^6^ cells and cultured during 12 h with media supplemented with 100 ng/ml of LPS (Sigma-Aldrich) or 250 μg/ml of synthetic hydroxyapatite nano-crystals (HA) (Sigma). At determined time points cells were harvested for RNA extraction and quantification of gene expression, as described below.

### Protein extraction

Leukocytes and cell lines total protein was obtained by extraction with RIPA buffer (50mM Tris HCl pH 8, 150 mM NaCl, 1% NP-40, 0.5% sodium deoxycholate, 0.1%SDS) for 1h at 4°C, with agitation, followed by a centrifugation at 16 000 xg for 15 min at 4°C. Protein concentration was assessed using Micro BCA kit (Thermo Scientific), according to the manufacturer’s instructions.

### Electrophoresis and Western blot

Aliquots of total protein extracts, ranging from 5 μg to 30 μg, were size separated in a 4–12% (w/v) gradient polyacrylamide precast gel containing 0.1% (w/v) SDS (NuPage, Invitrogen, Carlsbad, CA, USA)) and transferred onto a nitrocellulose membrane (Biorad, Richmond, CA, USA). Western blot detection of tGRP, cGRP, tMGP, cMGP and GAPDH was performed using specific primary antibodies CTerm-GRP (5 μg/ml), cGRP (1:1000 v/v) (GenoGla Diagnostics, Faro, Portugal), tMGP and cMGP (1:1000 v/v, both from VitaK BV, Maastricht, The Netherlands) previously validated as described [16, 30, 31, 33, 34, 36], and anti-GAPDH (1:500 v/v) (Santa Cruz Biotechnology). Detection was achieved using species-specific secondary horseradish peroxidase-conjugated antibodies and Western Lightning Plus-ECL (PerkinElmer Inc., Waltham, MA, USA). Image acquisition was obtained using an IQ LAS 4000 mini biomolecular imager (GE Healthcare, Lisbon, Portugal) and enhancement was performed with Adobe Photoshop. Semi-quantification was performed using ImageJ software, and relative protein levels normalized to GAPDH are presented as arbitrary units.

### RNA extraction

Total RNA was extracted from purified and cultured leukocytes (3 donors) and cell lines as described by Chomczynski and Sacchi [37]. Concentration of total RNA was determined by spectrophotometry at 260 nm and quality evaluated by agarose-denaturating gel electrophoresis.

### PCR analysis

One microgram of total RNA obtained from isolated leukocytes, THP-1 cell line, and 25 nanograms of total RNA isolated from exosomes were first treated with RQ1 RNase-free DNase (Promega, Madison, WI, USA) according to manufacturer’s instructions and reverse transcribed using Moloney-murine leukemia virus reverse transcriptase, RNase Out (both from Invitrogen, Carlsbad, CA, USA), and an oligo(dT) adapter (5’-ACGCGTCGACCTCGAGATCGATG (T)13–3’), according to manufacturer’s recommendations. PCR reactions and set up of technical replicates, were as follows: 5 μl of cDNA (from 1:10 or 1:5 cDNA synthesis), 0.6 μl of forward and reverse primers (0.3 μM final concentration) (Table 1) and 10 μl of SsoFast™ EvaGreen® Supermix (Bio-Rad) in reactions of 45 cycles. Fluorescence was measured at the end of each extension cycle in the FAM-490 channel and the melting profiles of each reaction were performed for monitoring of unspecific amplifications. Levels of gene expression were calculated using the comparative method (ΔΔCt) and normalized using gene expression levels of 18S or GAPDH housekeeping genes.

**Table 1:** Gene-specific primers used in this study.

Gene	Primer name	Sequence (5’ to 3’)
18S	18S_F	GGAGTATGGTTGCAAAGCTGA
	18S_R	ATCTGTCAATCCTGTCCGTGT
GAPDH	GAPDH_F	AAGGTGAAGGTCGGAGTCAACGGA
	GAPDH_R	TCGCTCCTGGAAGATGGTGATGGG
GRPF	GRPF1_F	GTCCCCCAAGTCCCGAGATGAGG
	GRPF1_R	CCTCCACGAAGTTCTCAAATTCATTCC
MGP	MGP_F	TGGAGGCTGGCACCTGATTTTG
	MGP_R	AAAAGGGGTGCAGCCAGACAAG
GGCX	GGCX_F	TTACACAGAGTCGGCGATGGAAGGAT
	GGCX_R	TACTGGATGTCAGGTCTGCGAAA
VKOR	VKOR_F	AGGGCAAGGCTAAGAGGCACTGAG
	VKOR_R	CTGGGCAATGGAAAGAGCTTTGGAGAC
IL-1β	IL-1β_F	TGGACAAGCTGAGGAAGATGCTGGT
	IL-1β_R	CCCTGGAGGTGGAGAGCTTTCAGTT
TNFα	TNFα_F	AGGGCCTGTACCTCATCTACTCCCA
	TNFα_R	AGCTGGAAGACCCCTCCCAGATAGA
NFkB	NFkB_F	GCAATCATCCACCTTCATTCTCAACTT
	NFkB_R	CCTCCACCACATCTTCCTGCTTAG
GRP(synthetic mRNA)	HSaGRPmMax_F*	GCTAATACGACTCACTATAGGGACAGGTC**ACCATG**ACTTGGAGACAGGCCGTCCTGCTGT
	HuGRP_Ex5R	ACACGGGGATGCCAATGGTGCTAC

*Underlined sequence, T7 Polymerase promoter minimal sequence; bold sequence, kozak sequence.

### Anti-inflammatory assays

Purified and characterized fully γ-carboxylated GRP from sturgeon (*A*. *Naccarii*) [29] and uncarboxylated recombinant human GRP produced in *E*. *Coli* [33], further referred as cGRP and ucGRP, respectively, were used in these assays as previously described [16, 31]. For the anti-inflammatory assays using LPS as inflammatory stimulating agent, THP-1 cells were seeded in 96 well plates at a density of 2.5 x10^5^ cells/well and differentiated as described above. After differentiation, media was removed and cells were treated for 24 h with 0.5, 0.75 and 1.5 μg/ml of cGRP and ucGRP, followed by addition of 50 ng/ml of LPS for additional 24 h. Dexamethasone treated cells (2 μM) were used as positive control. Conditioned media were collected and anti-inflammatory activity was analyzed using commercially available ELISA assays to measure TNFα (PreproTech) and PGE2 (Thermo Scientific) levels in cell supernatants, according to the manufacturer’s protocols. ELISA results were analyzed using a four parameter logistic curve fitting model in GraphPad Prism software.

For the anti-inflammatory assay using BCP crystals and protein mineral complexes (PMCs), BCP crystals were produced as previously described [16, 33] and sterilized overnight at 180°C to minimize endotoxins contamination. PMCs were prepared by incubating BCP crystals, resuspended in RPMI media at 100 μg/ml, with approximately 750 ng/ml of cGRP/ucGRP for 30 min at 37°C [16]. 100 μg/ml of BCP crystals and PMCs (PMC-cGRP and PMC-ucGRP), as well as 1.5 μg/ml of cGRP/ucGRP proteins alone, were added to differentiated THP-1 as described above, for a period of 24 h (controls consist of non-treated THP-1 MoM culture media). Conditioned media were collected and TNFα levels were measured using a commercially available ELISA (Thermo Scientific) according to the manufacturer’s protocol.

Cell viability of THP-1-MoM supplemented with 50 ng/ml LPS, 1.5 μg/ml of cGRP and ucGRP, 100 μg/ml of BCP crystals and PMC-cGRP/ucGRP, was determined at appropriate times using the CellTiter 96 cell proliferation assay (Promega, Madison, WI, USA), following manufacturer’s instructions.

### Overexpression with GRP mRNA

The pCRII-TOPO/GRP clone [34] was used as template to PCR amplify the complete GRP ORF using primers HSaGRPmMax_F, containing a T7 Polymerase promoter minimal sequence and a kozak sequence upstream ATG initiation codon, and HuGRP_Ex5R (Table 1). The resulting PCR product was used to *in vitro* synthetize capped and poly(A) tailed GRP mRNA using the mMESSAGE mMACHINE T7 Ultra kit (Ambion, Life Technologies), according to manufacturer’s recommendations. Final mRNA was recovered using Direct-zol RNA Miniprep kit (Zymo Research, Irvine, CA, USA), and quantified by spectrophotometry at 260 nm in a NanoDrop apparatus (Thermo Scientific). GRP mRNA transfection in THP-1 cells was performed with the jetMESSENGER^TM^ transfection reagent according to manufacturer’s instructions. Briefly, 100.000 cells in 500 μl of culture media were seeded per well in a 24-well plate 24 h before transfection. Transfection was performed using 0.5 μg mRNA/well following recommendations, and GRP gene expression analysis was performed in cells harvested for RNA extraction at 24, 48 and 72 h post-transfection by qPCR as described above. For anti-inflammatory assays, THP-1 cells were transfected as described, and after 24 h cells were stimulated with 50 ng/ml of LPS during 12 h, and 250 μg/ml of HA (Sigma) during 48 h. At determined time points, cells were harvested for RNA extraction and quantification of gene expression of the target inflammatory genes IL-1β, TNFα and NFkB, as described above (Table 1). Conditioned media were collected and TNFα levels were measured using a commercially available ELISA (PreproTech) according to the manufacturer’s protocol.

### Extracellular vesicles isolation

THP-1 were differentiated as described above with 25 ng/ml PMA in complete RPMI using exosome-depleted FBS [31] for 48 hours, followed by culture with serum starvation for another 20 hours. Conditioned medium was collected and EVs were isolated as described [31]. Briefly, EVs from media were fractionated through differential centrifugation at 2.000 xg for 15 min, 10.000 xg for 1h, 30.000 xg for 1h and 100.000 xg for another 2h. Recovered supernatant after 30.000 xg was filtrated through a 0.2 μm filter before centrifugation at 100.000 xg. All centrifugations were carried at 4°C. Final pellets were washed with PBS, pelleted again and ressuspended in PBS. Total RNA of isolated vesicles was obtained using Direct-zol RNA Miniprep kit (Zymo Research, Irvine, CA, USA), according to manufacturer’s instructions. Detection of CD9 (Invitrogen), tGRP and GAPDH was performed by Western blot as described above.

### Transmission Electron Microscopy (TEM)

EVs isolated by differential ultracentrifugation at 100.000 xg were adsorbed onto formvar-carbon coated grids and stained with 1% aqueous uranyl acetate. The grids were air dried before observation in a JEOL 1200EX transmission electron microscope.

### Statistical analysis

Data are presented as mean (n>2) ± standard error. Significance was determined for more than two groups using ordinary one-way ANOVA with either comparison between groups by Dunnett test or multiple comparisons with the Tukey’s test. Student’s t-test was used for comparison between two groups. The non-parametric Mann-Whitney U test was performed to confirm the difference of two group comparison. Statistical significance was defined as P< = 0.05 (*), P< = 0.01 (**), P< = 0.001 (***), and P<0.0001 (****).

## Results

### γ-carboxylated GRP and MGP are present in circulating leukocytes

**Fig 1:**
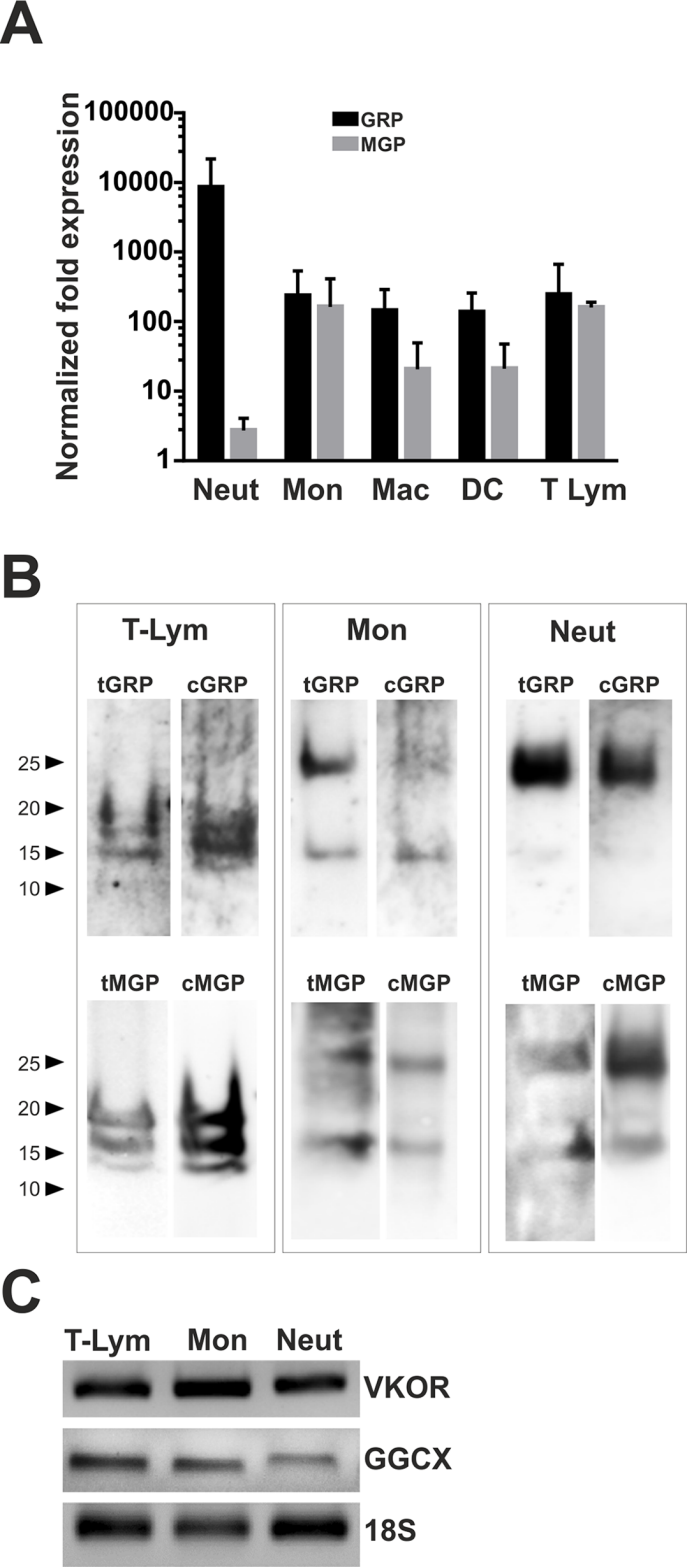
GRP and MGP are produced by leukocytes as γ-carboxylated proteins. Leukocyte subsets neutrophils (Neut), monocytes (Mon), and T lymphocytes (T-Lym) were isolated from human buffy coats, and macrophages (Mac) and dendritic cells (DC) were obtained by differentiation of isolated monocytes in culture. After purity assessment by flow cytometry, these isolated leukocyte populations were used to evaluate GRP and MGP production at gene and protein levels. (A) Relative fold expression (arbitrary units) of GRP and MGP determined by qPCR in isolated leukocytes. Means are presented as replicates of 3 biological healthy donors using 18S as housekeeping gene. (B) Total protein extracts of the isolated leukocyte populations were obtained with RIPA buffer and quantified using the Micro BCA kit. Five μg, ten μg and fifteen μg of total protein extracts from T Lym, Mon and Mac, respectively, were analyzed by Western blot to detect total GRP (tGRP) and total MGP (tMGP) protein forms using the validated CTerm-GRP and tMGP antibodies, and γ-carboxylated GRP (cGRP) and MGP (cMGP) protein forms using conformation-specific antibodies. Position of relevant molecular mass markers (kDa) is indicated on the left side. (C) Qualitative gene expression analysis of the γ-carboxylated related enzymes VKOR and GGCX by RT-PCR in T lymphocytes, monocyte and neutrophils. 18S amplification was used as loading control for sample integrity.

To investigate whether human leukocytes express the vitamin K-dependent protein GRP and their γ-carboxylation capacity, different human blood leukocyte subsets were isolated, and used to evaluate GRP gene expression and protein γ-carboxylation status. In addition, since MGP has been previously detected in macrophage foam cells *in vivo* [38], and shown to be up-regulated in RAW 264.7 mouse macrophage cells upon inflammatory stimulation with VSMCs conditioned media [6], prompted us to simultaneously study MGP as a related VKDP, potentially γ-carboxylated in leucocytes. Isolated leucocyte subsets of circulating neutrophils (Neut), monocytes (Mon) and T lymphocytes (T lym) were analyzed by flow cytometry and showed purity levels above 96% for each population (results not shown). Macrophages (Mac) and dendritic cells (DC) were obtained by differentiation from isolated monocytes, and cell differentiation was determined based on the observed gain of cell adherence (Mac) or dendritic (DC) under optic microscope observation (results not shown). Gene expression analysis by qPCR revealed the presence of both GRP and MGP mRNA in all leukocytes subsets tested (Fig 1A). In order to confirm the presence of GRP and MGP at the protein level in the major circulating leukocytes: monocytes, T lymphocytes and neutrophils, total protein extracts were analyzed by Western blot using previously validated antibodies for total GRP and MGP, and conformation specific antibodies for the detection of the specific γ-carboxylated forms [16, 30, 31, 33, 34, 36]. Positive GRP and MGP signals were detected in all the three cell subsets, using CTerm-GRP and tMGP antibodies, respectively (Fig 1B). Interestingly, different migration patterns were observed among the different leukocytes cell subsets for both GRP and MGP. While positive signal in monocytes (Mon) and neutrophils (Neut) was predominantly observed at 15 and 25 kDa, T lymphocytes (T-Lym) shown three positive bands ranging from 14 kDa to 20 kDa for both GRP and MGP (Fig 1B). Since the presence of Gla residues in these VKDPs has been demonstrated as a functional requirement, detection of γ-carboxylated GRP and MGP in the leukocytes subsets was further performed using conformational-specific cGRP and cMGP antibodies, respectively (Fig 1B). Positive signals were detected for both cGRP and cMGP with similar signal profiles to those obtained with antibodies for the total protein (tMGP and tGRP) (Fig 1B). Protein integrity on the RIPA extracts were evaluated by CBB staining after SDS-PAGE analysis, and detection of GAPDH by Western blot (results not shown). A similar detection pattern of GRP with multiple positive bands, using the CTerm-GRP and cGRP antibodies, was previously obtained in protein extracts from calcified aortic valves, where GRP identity was further confirmed by LC-MS/MS [31]. Since γ-carboxylation reaction machinery is a multi-component enzyme dependent reaction that includes VKOR and GGCX enzymes, the presence of these genes was also evaluated. Gene expression for both enzymes was confirmed in all the circulating leukocytes cell subsets analyzed (Fig 1C), further corroborating the capacity of these human leukocyte cells to γ-carboxylate GRP and MGP.

### THP-1 cell line is a suitable model for studying VKDPs in leukocytes

**Fig 2:**
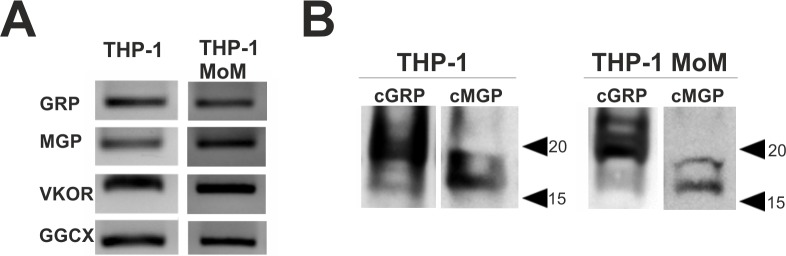
γ-carboxylated GRP and MGP are produced in THP-1 cell line. THP-1 and THP-1 MoM differentiated with 25 ng/ml of PMA during 48h were cultured in control conditions and harvested for RNA and protein extraction. (A) Qualitative gene expression analysis of GRP, MGP, VKOR and GGCX by RT-PCR in undifferentiated THP-1 representing monocyte cells (THP-1), and in differentiated THP-1 representing macrophages (THP-1 MoM). (B) Western blot analysis of thirty μg of total RIPA protein extracts of THP-1 and THP-1 MoM cells using the conformation-specific antibodies recognizing γ-carboxylated GRP (cGRP) and MGP (cMGP). Position of relevant molecular mass markers (kDa) is indicated on the right side.

Since monocytes and macrophages are key players during inflammation and particularly associated to calcification related chronic inflammatory diseases [1–5], we decided to assess if THP-1 monocytic cell line could be used for further studies involving the association of GRP with inflammatory processes. The protein and gene profiles of GRP and MGP were analyzed and compared with its biological human cell counterpart. The presence of mRNAs for GRP, MGP, as well as for the γ-carboxylation related enzymes GGCX and VKOR, was detected by RT-PCR in both THP-1 and differentiated macrophage THP-1 (THP-1 MoM) (Fig 2A). Western blot analysis of THP-1 and THP-1 MoM protein extracts shown detection of γ-carboxylated GRP and MGP in both cell cultures, with similar patterns among monocytic and macrophagic states (Fig 2B). However, dissimilar migration profiles for both cGRP and cMGP were found between THP-1 (Fig 2B) and biological monocytic cells (Fig 1B), which might be related to different γ-carboxylation degrees reflecting putative differences between γ-carboxylation capacity of the *in vivo* and *in vitro* systems [29]. Nevertheless, the production of γ-carboxylated GRP in THP-1 and THP-1 MoM cells indicates the suitability of using these cells to further study the involvement of GRP in monocytes/macrophages inflammatory mechanisms.

### GRP and MGP are up-regulated in LPS- stimulated THP-1 monocytes and macrophages

**Fig 3:**
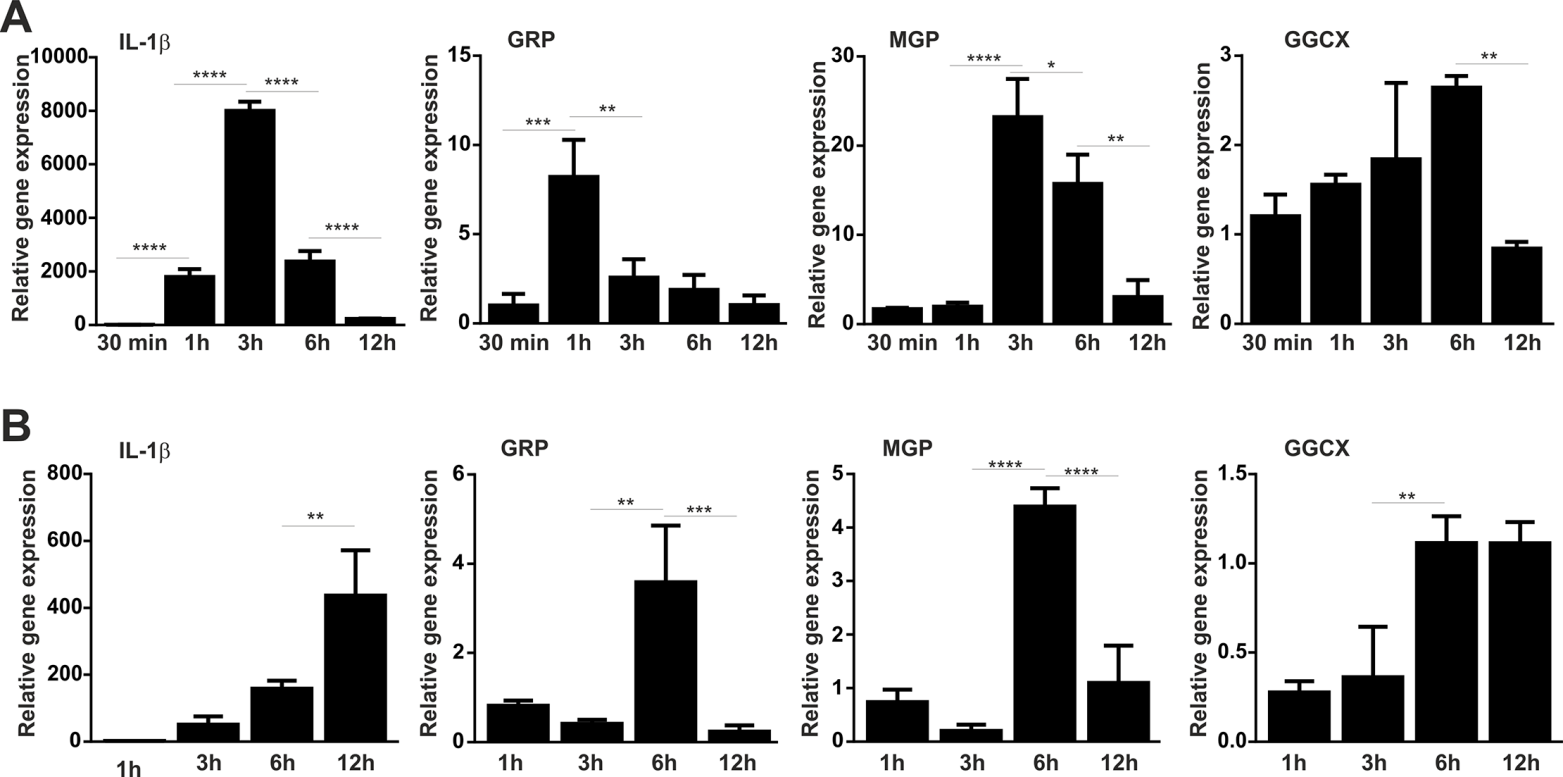
GRP and MGP are up-regulated in LPS stimulated THP-1 cells. THP-1 and differentiated THP-1 macrophage cells were stimulated with 100 ng/ml of LPS and harvested for RNA extraction at determined time points during 12h. Relative gene expression analysis of the inflammatory marker IL-1β, GRP, MGP and GGCX was determined by qPCR in monocytes (THP-1 cells) (A) and in differentiated THP-1 macrophages (B) at indicated time points. Gene expression levels are relative to the expression of control untreated cells during each time point. GAPDH was used as housekeeping gene and data is presented as means (n = 4) ± standard error of duplicates of two independent experiments. Ordinary one-way ANOVA was used and multiple comparisons were achieved with Tukey's test. Statistical significance was defined as P< = 0.05 (*), P< = 0.01 (**), P< = 0.001 (***) and P<0.0001 (****).

Since GRP has been previously associated with inflammatory events in chondrocytes and synoviocytes [16], and MGP was recently shown to be up-regulated, together with IL-1β, in RAW 264.7 cells after treatments with VSMCs conditioned media [6], we studied the involvement of GRP and MGP in monocytes/macrophages inflammatory responses using undifferentiated and differentiated THP-1 cell line, stimulated with LPS. Maximum inflammatory response of THP-1 monocytes to LPS was detected after 3 h by significant up-regulation of IL-1β, followed by a progressive decrease until 12 h (Fig 3A). The pattern of GRP maximum level of expression was obtained just 1 h after LPS stimulation, progressively decreasing until control levels at 12 h, while MGP level of expression was found similar to IL-1β, with an expression peak at 3 h (Fig 3A). Interestingly, only a slight but gradual increase in GGCX expression was found during stimulation, with levels close to non-stimulated cells until 6 h, and a significant decrease at 12 h (Fig 3A). The inflammatory response of THP-1 macrophages appeared to be delayed compared to that of THP-1 monocyte cells, as previously reported [39] with maximum up-regulation of IL-1β only at 12 h after LPS stimulation (Fig 3B). GRP and MGP expression profiles in macrophages were found highly similar, with a peak detected at 6 h, and a significant decrease at 12 h, while an up-regulation of GGCX was found at 6 h and maintained until 12 h with similar levels (Fig 3B). These results indicate that GRP and also MGP are involved in the endogenous inflammatory response mechanisms of monocytes and macrophages. In addition, a correlation between increased levels of GRP and MGP, and up-regulation of GGCX was observed in macrophages, suggesting the involvement of γ-carboxylation in the inflammatory mechanism mediated by VKDPs such as GRP and MGP.

### Hydroxyapatite induces a pro-inflammatory response of THP-1 macrophages and an up-regulation of GRP and MGP

**Fig 4:**
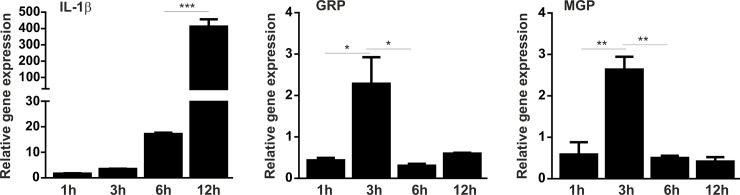
GRP and MGP are up-regulated in hydroxyapatite stimulated THP-1 MoM cells. Differentiated THP-1 macrophage cells were stimulated with 250 μg/ml of synthetic hydroxyapatite nano-crystals and harvested for RNA extraction at determined time points during 12h. Relative gene expression analysis of the inflammatory marker IL-1β, GRP and MGP was determined by qPCR at indicated time points. Gene expression levels are relative to the expression of control untreated cells during each time point. GAPDH was used as housekeeping gene and data is presented as means (n = 3) ± standard error of duplicates of two independent experiments. Ordinary one-way ANOVA was used and multiple comparisons were achieved with Tukey's test. Statistical significance was defined as P< = 0.05 (*), P< = 0.01 (**), and P< = 0.001 (***).

To determine the effect of hydroxyapatite (HA) on macrophage cells and the corresponding expression patterns of GRP and MGP, synthetic HA nano-crystals were used to stimulate THP-1 MoM cells over a 12 h period. Progressive increase in IL-1β indicate that HA induced a pro-inflammatory response of THP-1 MoM cells, while up-regulation of GRP and MGP was observed at 3 h of HA treatment (Fig 4).

### GRP decreases the pro-inflammatory response of LPS-stimulated macrophagic THP-1 cells

**Fig 5:**
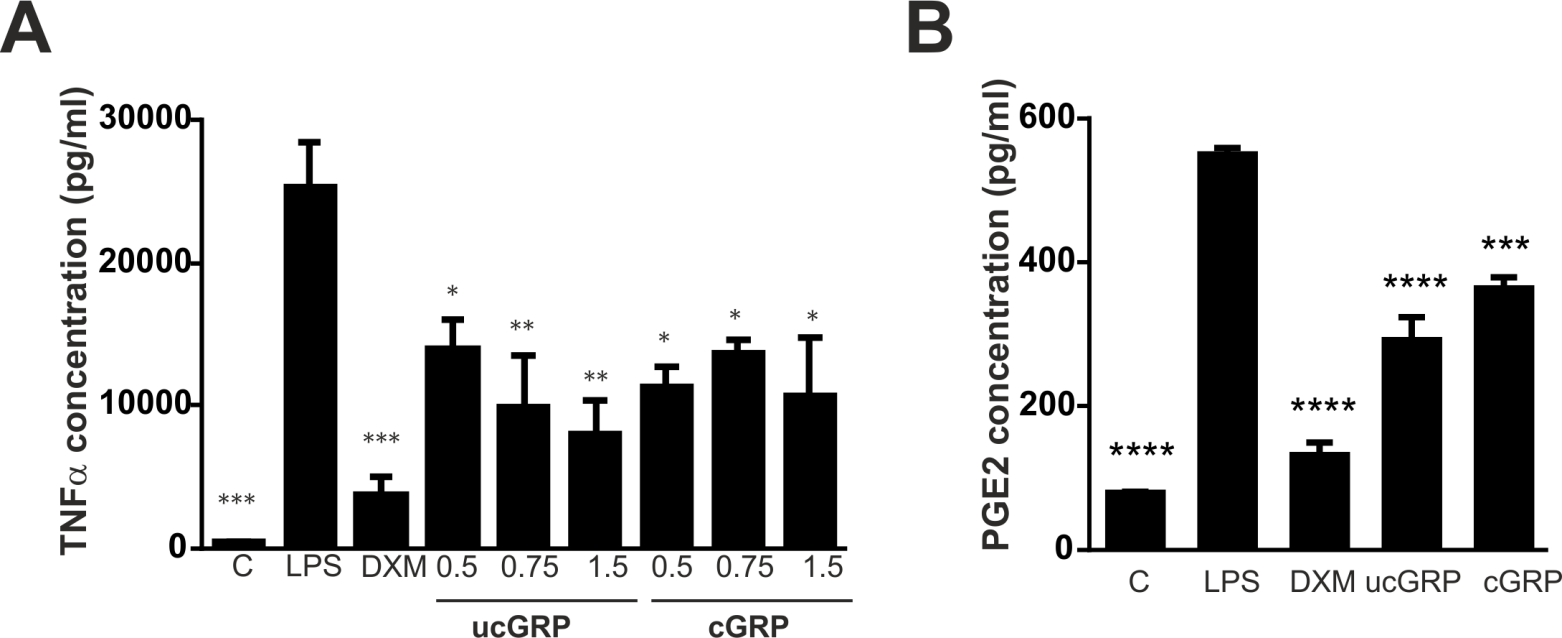
GRP reduces TNFα and PGE2 production in THP-1 MoM cells stimulated with LPS. (A) Differentiated THP-1 MoM cells were treated with 0.5 μg/ml, 0.75 μg/ml and 1.5 μg/ml of purified cGRP and ucGRP proteins for 24 h, followed by exposure to 50 ng/ml LPS for additional 24 h. Cells treated with 2 μM dexamethasone (DXM) were used as a positive anti-inflammatory control, and non-stimulated cells (C) as controls to LPS stimulation. Conditioned cell culture media were collected and used to determine TNFα accumulation by ELISA assays. Data are presented as means (n = 3) ± standard error of triplicates of two independent experiments. Ordinary one-way ANOVA was used and multiple comparisons were achieved with Dunnett's test. Statistical significance was defined as P< = 0.05 (*), P< = 0.01 (**), and P< = 0.001 (***). (B) Differentiated THP-1 MoM cells were treated with 1.5 μg/ml of purified cGRP and ucGRP proteins for 24 h and exposed to LPS as described in (A), and PGE2 accumulation was determined in conditioned media through ELISA assays. Data are presented as means (n = 2) ± standard error of replicates of two independent experiments. Ordinary one-way ANOVA was used and multiple comparisons were achieved with Dunnett's test. Statistical significance was defined as P< = 0.001 (***) and P<0.0001 (****).

Our results showing a correlation between GRP and GGCX expression in macrophages (Fig 3B), suggest the involvement of γ-carboxylation in the inflammatory response. In order to further determine if GRP could have a direct effect on the LPS-stimulated immune function of THP-1 cells and to further unveil the modulatory effect of its γ-carboxylation state, THP-1 MoM cells were treated with increasing concentrations of purified cGRP/ucGRP previously used in functional assays [16, 31], or dexamethasone (DXM) during 24 h, followed by exposure to LPS. Inflammatory responses were determined by measuring TNFα accumulation in cell media (Fig 5A). When compared to the control condition, LPS stimulated cells showed a significant increase in TNFα production while cells treated with the control anti-inflammatory agent DXM showed a significant decrease in the levels of TNFα. Treatments with ucGRP resulted in a dose-dependent decrease of TNFα levels relative to LPS treated cells, with a maximum decrease achieved at 1.5 μg/ml of ucGRP (Fig 5A). Treatments with cGRP also shown decreased TNFα levels although to a less extent and independent of the concentrations used (Fig 5A). These results were further confirmed by measuring the PGE2 production in a similar experiment setting using 1.5 μg/ml of ucGRP/cGRP (Fig 5B). Again, the highest inhibitory effect on PGE2 production was obtained with ucGRP when compared with the same treatments using cGRP (Fig 5B). THP1-MoM cell viability was unaffected in the presence of LPS and ucGRP or cGRP at 1.5 μg/ml (results not shown).

### Coating of BCPs with GRP attenuates the pro-inflammatory response of THP-1 macrophages

**Fig 6:**
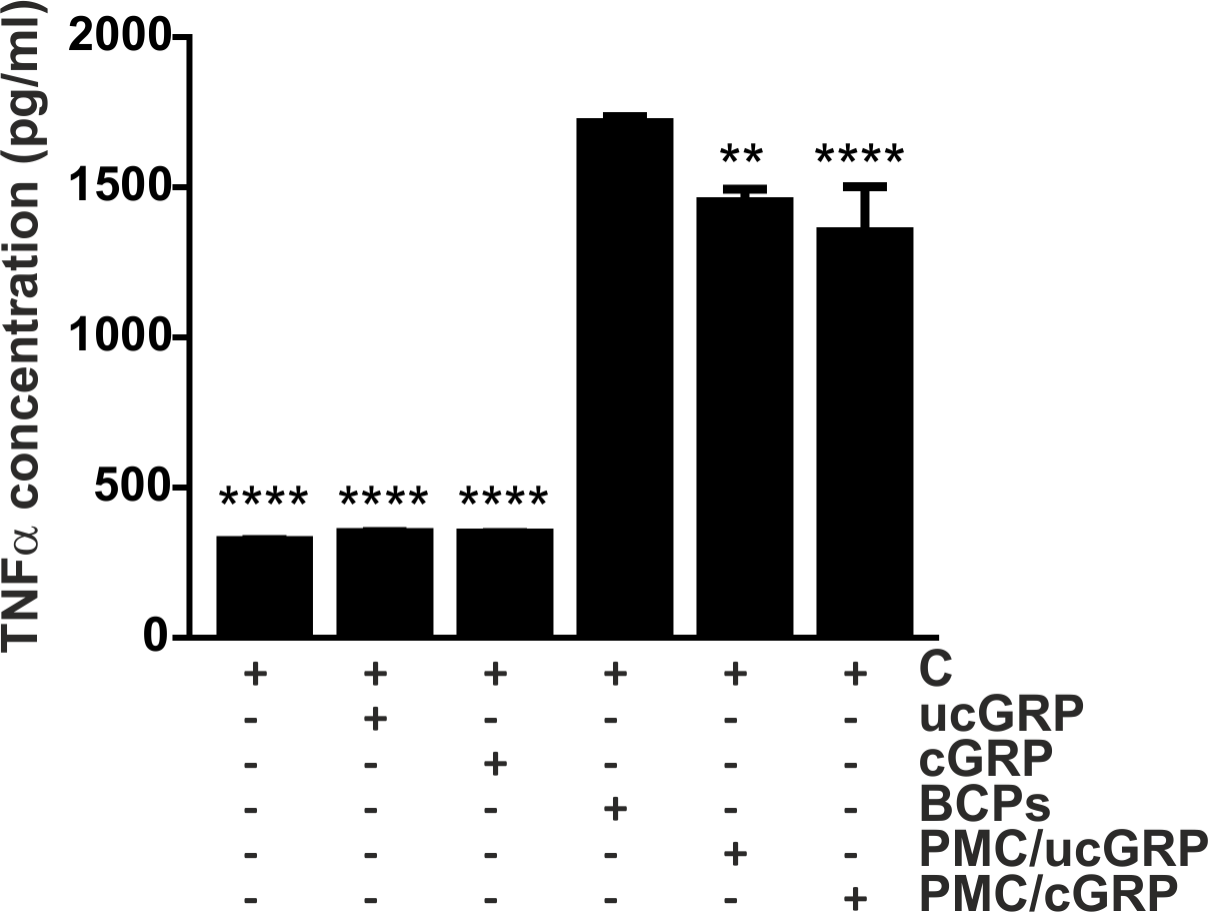
Coating of BCPs with GRP reduces TNFα production in THP-1 MoM when compared with naked crystals. Differentiated THP-1 MoM cells were treated with 100 μg/ml of BCP crystals or BCPs-coated with cGRP/ucGRP (PMC/cGRP; PMC/ucGRP) proteins for 24 h, and non-stimulated cells were used as control (C). Control non-stimulated cells were also treated with 1.5 μg/ml of cGRP/ucGRP proteins for comparison. Conditioned media were collected and used to determine TNFα accumulation through ELISA assays. Data are presented as means (n = 3) ± standard error of triplicates of two independent experiments. Ordinary one-way ANOVA was used and multiple comparisons were achieved with Dunnett's test. Statistical significance was defined as P< = 0.01 (**) and P<0.0001 (****).

Since BCPs coated with GRP were able to decrease the inflammatory response of chondrocytes and synoviocytes when compared to naked crystals [16], we studied the effect of BCPs coated with either cGRP/ucGRP (PMCs) in the macrophage THP-1 pro-inflammatory reaction. As expected, BCP crystals induced the production of TNFα when compared to non-stimulated cells (Fig 6). However, PMCs containing either cGRP or ucGRP resulted in a decrease on TNFα levels and interestingly, the highest effect was observed with PMCs-cGRP (Fig 6). As a control, cGRP/ucGRP were added to the culture media of control non-stimulated cells and showed no effect on TNFα production (Fig 6). THP1-MoM cell viability was unaffected in the presence of tested BCPs and PMC-ucGRP or PMC-cGRP (results not shown).

### GRP overexpression decreases THP-1 inflammatory response to LPS and HA stimulation

**Fig 7:**
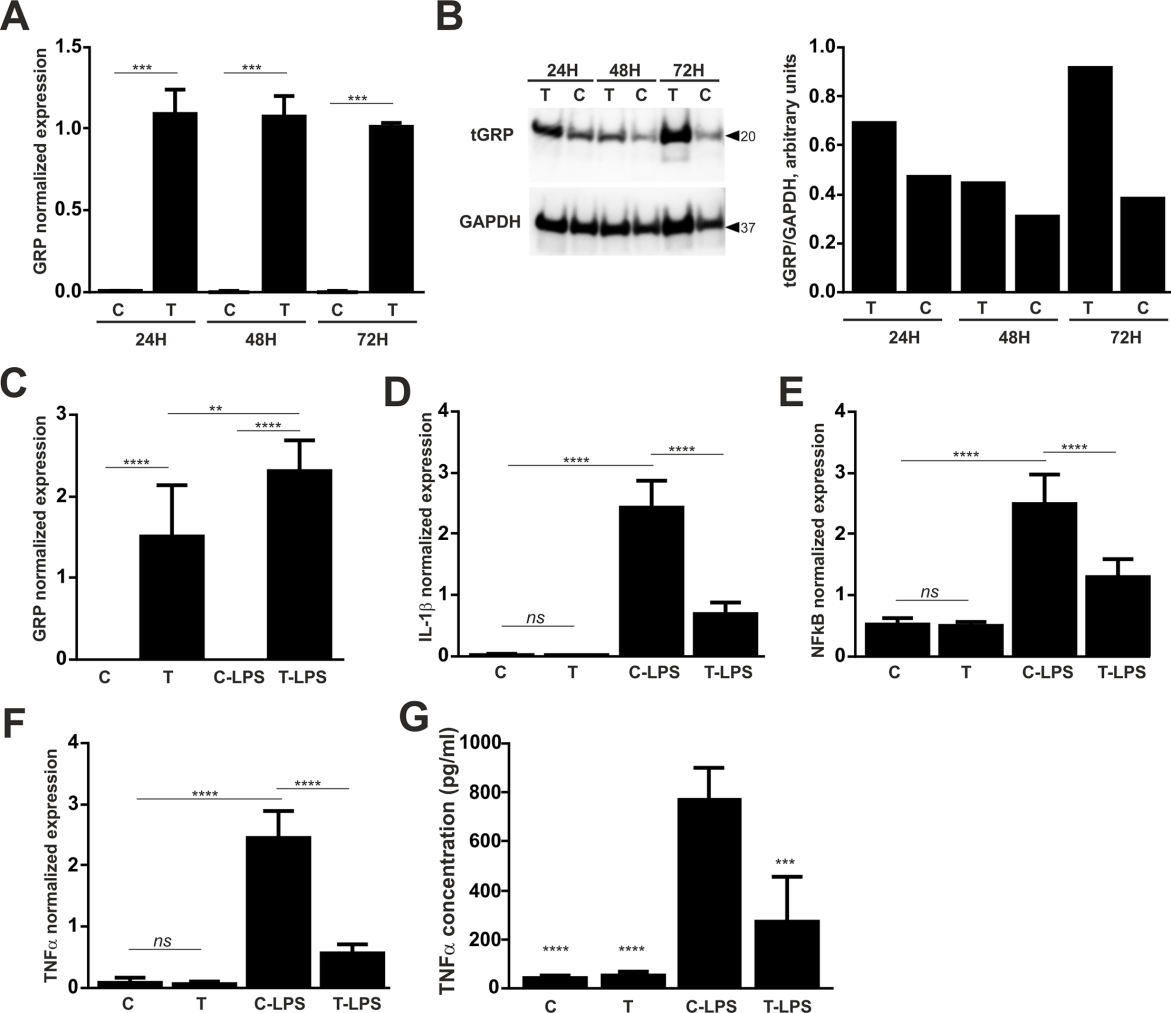
Overexpression of GRP in THP-1 cells rescues LPS induced inflammation. THP-1 cells were transiently transfected with GRP mRNA during 24, 48 and 72 h, and GRP overexpression was evaluated by measuring levels of GRP mRNA (A) and protein production (B). (A) GRP Normalized expression was determined by qPCR in non-transfected (C) and transfected (T) cells at the indicated time points. Data are presented as means (n = 3) ± standard error of duplicates of two independent experiments. Student’s t-test was used for comparison between C and T groups at each time point. Statistical significance was defined as P< = 0.001 (***). (B) Twenty micrograms of total protein extracts from the conditions described above were analysed by Western blot to detect tGRP and GAPDH proteins, and relative protein levels normalized to GAPDH were obtained using ImageJ software and are presented as arbitrary units. (C-G) Transfected (T) and non-transfected THP-1 (C) cells were cultured for 24 h and either maintained in control conditions (C, T) or stimulated with LPS (C-LPS, T-LPS) for additional 12 h. Normalized GRP gene expression was determined by qPCR (C), and inflammatory response was evaluated by measuring gene expression of the inflammatory markers IL-1β (D) NFkB (E) and TNFα (F) and TNFα accumulation in cell culture media by ELISA (G). (C-F) Data are presented as means (n = 6) ± standard error of triplicates of two independent experiments. Ordinary one-way ANOVA was used and multiple comparisons were achieved with Tukey's test. Statistical significance was defined as P< = 0.01 (**), P< = 0.001 (***) and P<0.0001 (****). *ns*, non-significant. (G) Data is presented as means (n = 4) ± standard error of triplicates of two independent experiments. Ordinary one-way ANOVA was used and multiple comparisons were achieved with Dunnett's test. Statistical significance was defined as P< = 0.001 (***) and P<0.0001 (****).

To further study the anti-inflammatory capacity of endogenous GRP, THP-1 cells were transiently transfected with *in vitro* transcribed GRP mRNA. To determine the time required to achieve GRP overexpression, a time course study was performed at 24, 48 and 72 h post-transfection. Quantification of GRP mRNA levels by qPCR shown sustained overexpression from 24 to 72 h (Fig 7A), and Western blot analysis confirmed increased protein accumulation in transfected cells (Fig 7B). Non-transfected (C) and transfected (T) THP-1 cells were then cultured in control conditions and stimulated with LPS [non-transfected cells stimulated with LPS (C-LPS); transfected cells stimulated with LPS (T-LPS)]. While overexpression of GRP was confirmed in control transfected cells, levels of GRP mRNA were further upregulated upon stimulation with LPS (Fig 7C). The inflammatory response in the presence of increased intracellular levels of GRP was evaluated through gene expression of inflammatory marker genes and measurements of TNFα accumulation in conditioned media. Down-regulation of the inflammatory markers IL-1β and NFkB were found in transfected cells when stimulated with LPS relative to non-transfected cells (Fig 7D and 7E). Levels of TNFα at gene (Fig 7F) and protein (Fig 7G) levels, were found similar in non-transfected and transfected THP-1 cells cultured in control conditions, while LPS-stimulated cells shown decreased levels of TNFα in transfected cells when compared to non-transfected.

**Fig 8:**
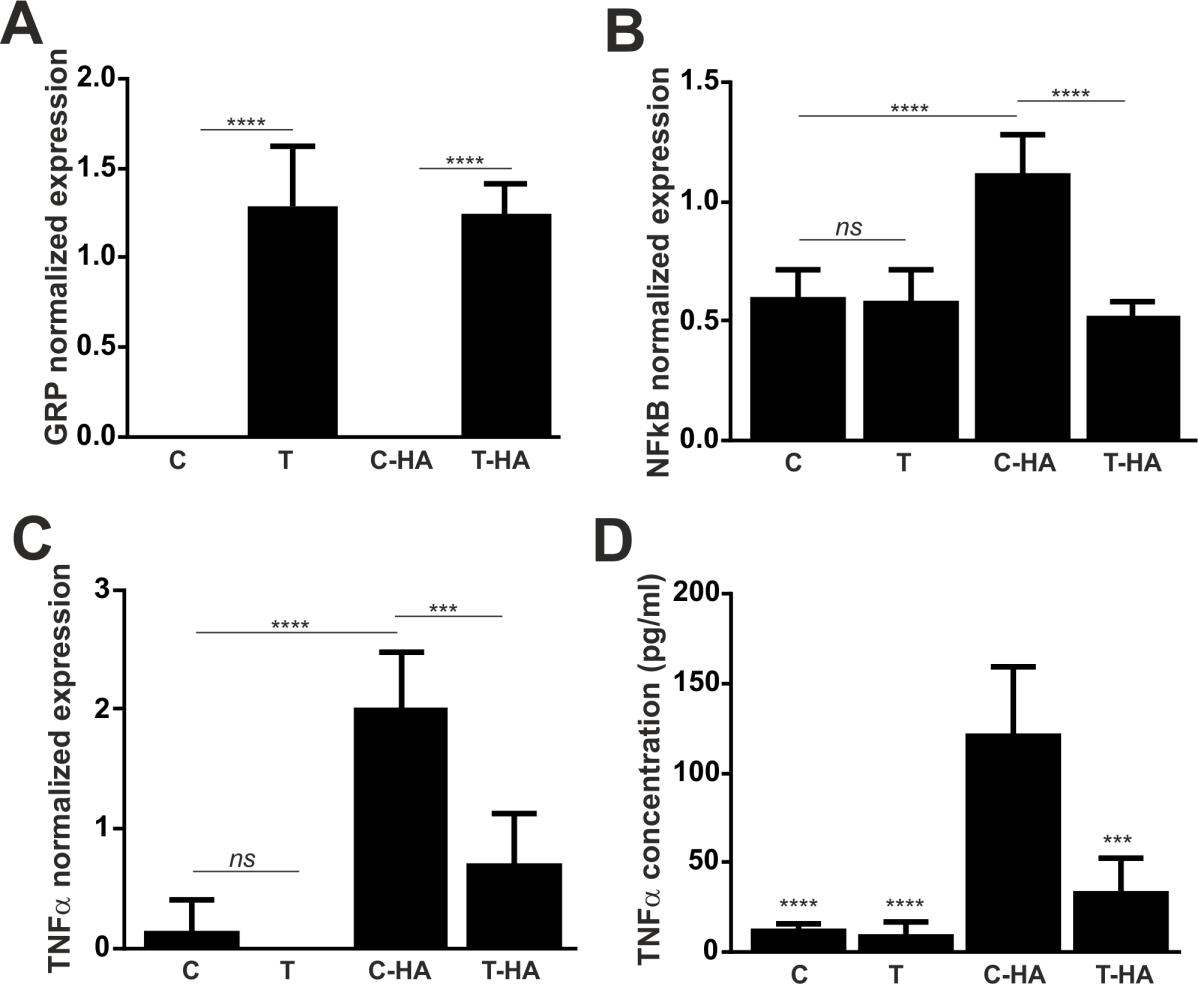
Overexpression of GRP in THP-1 cells rescues hydroxyapatite induced inflammation. THP-1 cells were transiently transfected with GRP mRNA for 24 h and either maintained in control conditions (C, T) or stimulated with HA (C-HA, T-HA) for additional 48 h. Gene expression of GRP (A) and the inflammatory marker genes NFkB (B) and TNFα (C) were determined by qPCR (A), and levels of TNFα accumulation were measured by ELISA in conditioned culture media (D). (A-C) Data are presented as means (n = 6) ± standard error of triplicates of two independent experiments. Ordinary one-way ANOVA was used and multiple comparisons were achieved with Tukey's test. Statistical significance was defined as P< = 0.01 (**), P< = 0.001 (***) and P<0.0001 (****). *ns*, non-significant. (D) Data are presented as means (n = 4) ± standard error of triplicates of two independent experiments. Ordinary one-way ANOVA was used and multiple comparisons were achieved with Dunnett's test. Statistical significance was defined as P< = 0.001 (***) and P<0.0001 (****).

Stimulation of THP-1 cells with HA shown similar overexpression levels of GRP mRNA in non-stimulated and stimulated transfected cells (Fig 8A), while down-regulation of NFkB and TNFα gene expression was observed in HA stimulated THP-1 overexpressing GRP (Fig 8B and 8C). In concordance, decreased levels of secreted TNFα were obtained in transfected cells stimulated with HA when compared to stimulated non-transfected cells (Fig 8D).

These results shown that increased expression of GRP is capable of decreasing the inflammatory responses of THP-1 cells induced by LPS and HA, acting as an endogenous anti-inflammatory and protective agent.

### GRP is present in extracellular vesicles secreted by THP-1 macrophages

**Fig 9:**
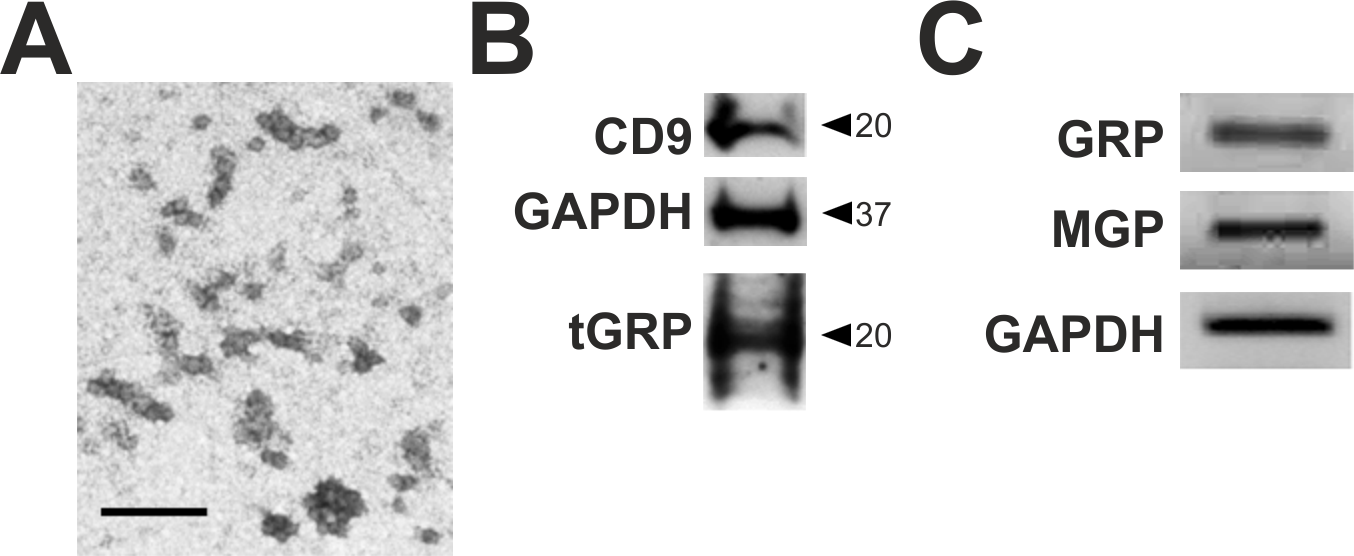
GRP is present in THP-1 MoM extracellular vesicles (EVs) at protein and mRNA levels. EVs were isolated from THP-1 MoM cell conditioned media by differential ultracentrifugation at 100.000 xg and characterized by TEM (A), Western blot for the exosomal marker CD9, GAPDH and total GRP (tGRP) (B), and qualitative analysis of GRP, MGP and GAPDH mRNA (C). Scale bar in panel (a) represents 200 nm.

Since extracellular vesicles (EVs) are known to be released by macrophages [13], and GRP was recently shown to be present in EVs derived from VSMCs and implicated in the vascular calcification process [31], we further explored the association of GRP with THP-1 MoM released EVs. EVs isolated from serum-starved THP-1 MoM through differential ultracentrifugation at 100.000 xg (100K) were characterized by TEM and Western blot analysis (Fig 9A and 9B). TEM analysis of the 100K population showed a homogeneous population of small particles with an average size of 30 nm (Fig 9A) and positive for cluster of differentiation 9 (CD9) and GAPDH, as determined by Western blot (Fig 9B). Interestingly, GRP was detected in the 100K isolated EVs both at protein (Fig 9B) and mRNA levels (Fig 9C). The detected protein pattern was similar to that of THP-1 MoM cell lysates (Fig 2B) with a predominant band at 20 kDa, and the presence of GRP and MGP mRNA in EVs was qualitatively determined by RT-PCR of EVs isolated total RNA (Fig 9C).

## Discussion

1. In this work, we demonstrated that GRP and MGP are expressed and translated by human leukocytes subsets as γ-carboxylated vitamin K-dependent proteins, which can be released into circulation or peripheral tissues, as extracellular vesicles components. GRP was shown to be involved in the inflammatory mechanism response, functioning as an endogenous and exogenous anti-inflammatory agent in THP-1 and THP-1 MoM stimulated cells. Interestingly, while γ-carboxylation can be an active process in immune cells, this post-translation modification doesn’t seem to be a critical factor for the GRP anti-inflammatory activity.

GRP has been associated with mineralization processes in several ectopic calcification-associated diseases [16, 30, 31, 33, 34], and shown to act as a calcification inhibitor in vascular and articular tissues [31, 16]. More recently, we have shown that GRP is also involved in inflammatory processes in osteoarthritis, acting as an anti-inflammatory agent in both chondrocytes and synoviocytes [16], although its involvement in the immune cells inflammatory response still remained unknown. The presence of GRP in all leukocytes subsets tested suggests that it is a constitutive component of immune cells and might be involved at different levels in the human immune system. In concordance, GRP has been previously detected in foam macrophages in calcific aortic valve disease [31], and co-localized with CD45 in osteoarthritic synovial membrane, characterized by lymphocytes and plasma cells infiltration [16]. Since in a context of ectopic calcification-dependent diseases, such as atherosclerosis and osteoarthritis, monocytes and macrophages are key players in disease initiation and progression, our focus was given to study the role of GRP in these particular immune cells. For that, we have used the THP-1 cell line that is the most widely used model for *in vitro* studies of primary human monocytes/macrophages, allowing the investigation on the function and regulation of monocytes and macrophages, overcoming the experimental limitations of using non-proliferative and short life spanning human primary macrophages [40]. As recently proposed, the function of GRP as a mediator factor linking mineralization and inflammatory processes might not be exclusively mediated by mineralization events [16]. Here, using the THP-1 monocytes/macrophages cell line, we shown that GRP expression is up-regulated under inflammatory stimulation with both LPS and hydroxyapatite. Also, treatments of THP-1 macrophage cells with purified cGRP/ucGRP or with BCPs coated with either cGRP/ucGRP (PMCs), resulted in decreased levels of TNFα or PGE-2 production. Moreover, GRP overexpression is able to counteract the inflammatory response of THP-1 when stimulated with either LPS or HA. These results strongly indicate an anti-inflammatory function of GRP, acting not only as an exogenous agent but also as an endogenous factor involved in inflammatory response mechanisms and providing a protective effect. Calcification and inflammation processes are widely known to be intimately correlated, with macrophages playing a key role due to their contribution to matrix degradation and calcification through the release of pro-inflammatory cytokines, prostaglandins and/or production of osteogenic factors [2, 6, 13, 19, 41, 42]. The accumulation of macrophages within the vascular wall has been regularly co-localized with calcium deposits and shown to have an important role in the initiation of atherosclerosis development and vascular calcification [12, 13]. Soluble factors produced by activated macrophages have been shown to promote osteochondrogenic differentiation of VSMCs increasing the production of osteoblast-like factors such as BMP-2 and decreasing mineralization inhibitors such as MGP, ultimately leading to increased calcium mineral deposition [2, 6]. On the other hand, micro-calcifications have been proposed to be involved in macrophage recruitment in early stages of atherosclerosis [43], and BCP crystals shown to stimulate the production of pro-inflammatory cytokines by macrophages, which affect VSMCs differentiation, in a calcification-inflammation pathological feeding cycle [19 and described in detail previously [44]]. In this context it is conceivable that GRP produced by monocytes/macrophages at sites of calcification might contribute to the high levels of protein accumulation previously detected at sites of mineral deposition [16, 31]. Moreover, the GRP anti-mineralization function described as a direct effect of the protein as a modulator of crystal formation and/or growth [16], could also be related with its anti-inflammatory action, by decreasing macrophage cytokine production and consequent decrease in cell differentiation and calcification events at tissue and cell levels. In concordance, GRP was shown to mediate monocyte and macrophage pro-inflammatory responses by decreasing the production of inflammatory mediators such as TNFα, PGE2 and IL1β, and the transcription factor NFkB. The release of TNFα by activated macrophages has long been reported to affect atherosclerosis, VSMCs osteogenic activity and calcification, exerting multiple effects during atherogenesis, including increasing permeability of endothelial cells, promoting monocyte adhesion, inducing macrophage differentiation, and promoting foam cell formation. Inhibition of TNFα in ApoE(-/-) mice was shown to reduce by 50% the size of aorta lesions [45]. TNFα has a pivotal role in enhancing VSMCs calcification through the activation of NFκB, leading to a decreased expression of ankyloses protein homolog (ANKH) that controls pyrophosphate (PPi) efflux, accompanied by a reduction in PPi export [46]. In addition, TNFα and IL-1β are reported to enhance VSMCs osteogenicity by increasing BMP-2 and reducing MGP expression [6]. Prostaglandin E2 (PGE2) has been implicated in the instability of atherosclerotic plaques and COX-2 expression was found to be up-regulated in human aortic valve interstitial cells from stenotic valves than in control AVICs [47, 48]. Also, the production of catabolic and pro-inflammatory mediators, including cytokines, chemokines, cyclooxygenase-2 (COX-2), PGE2 and inducible nitric oxide synthase (NOS2), is widely reported to promote the destruction of the cartilage matrix and joint function loss in OA [49]. In this line, we propose that GRP is involved in the feed-back loop between inflammation and calcification, as a molecular link between macrophage-released osteogenic factors and calcification-related agents modulating inflammation.

Interestingly, although γ-carboxylation of GRP has been shown as essential for its role as mineralization inhibitor [16, 31], its anti-inflammatory activity was found to be independent of its γ-carboxylation status in articular tissues [16]. Similarly, our results show decreased levels of TNFα and PGE2 in THP1-MoM cells treated with either cGRP/ucGRP proteins, or PMCs-cGRP/PMCs-ucGRP, but the significance of its γ-carboxylation in inflammatory reactions is presently unclear, and should be further evaluated. Despite the widely accepted anti-inflammatory effect of vitamin K [21–23, 27], the role and involvement of VKDPs in immune cells has been poorly explored. Dietary supplementation of vitamin K were shown to suppress LPS-induced inflammation [21], and vitamin K status has been associated with lower concentrations of inflammatory markers *in vivo* [22, 27]. *In vitro*, vitamin K has been associated with decreased production of pro-inflammatory cytokines, such as TNFα, IL-1β, IL-6, and osteoprotegerin, and proposed to be mediated via the inactivation of NFkB signalling pathway, independent of its Gla formation activity [23, 27]. These *in vitro* studies not only suggest a direct effect of vitamin K, not related with the carboxylation of VKDPs, but also propose that the anti-inflammatory mechanism is associated with the vitamin K protective effect against oxidative stress [27]. In this study, we have demonstrated that γ-carboxylation-related machinery such as GGCX and VKOR genes are expressed in major circulating leukocytes. Moreover, using previously validated conformational-specific antibodies [16, 30, 31, 33, 34, 36], we were able to show that γ-carboxylated GRP and MGP are produced by these immune cells. Nevertheless, controversial data has been reported concerning the effect of warfarin on the immune system, described to act as an anti-inflammatory, pro-inflammatory, or not related agent [27, 50, 51]. Altogether, these data clearly emphasize the need for additional molecular studies to unveil the activity of GRP in the inflammatory process and further evaluate the modulating potential of its γ- carboxylation status.

Increased concentrations of calcium and phosphate were shown to induce macrophage release of matrix vesicles (MVs) with high calcification potential that participate in the initiation of microcalcification [13]. γ-carboxylation of GRP and MGP might be associated with macrophage action on calcification through the recently proposed mechanisms involving the release of calcifying MVs [13]. Our results have shown that THP-1 MoM extracellular vesicles (EVs), similarly obtained by differential ultracentrifugation at 100.000 xg, contain GRP. We had previously demonstrated that GRP is a new player in the mineralization competence of VSMCs derived-EVs from aortic segments [31]. In fact, the release of EVs capable of efficiently nucleate hydroxyapatite has been demonstrated as a key event in the initiation of both physiological and pathological calcification in VSMCs and chondrocytes [8, 10, 52]. Under normal conditions, VSMCs-derived EVs do not calcify because of their loading with mineralization inhibitors acting to block mineral nucleation, and loss of functional mineralization inhibitors such as GRP, cMGP and fetuin A is crucial to promote EVs calcification [8, 10, 31, 52]. γ-carboxylation of MGP and GRP has been demonstrated as crucial for adequate mineralization inhibition in both VSMCs and chondrocytes, and increasing undercarboxylated MGP accumulation promote EVs mineralization [16, 31, 10, 52]. Interestingly, foam cells found at regions of lipid accumulation in atheromas surrounding areas in calcified aortic valve disease, mainly produce ucGRP [31]. The presence of GRP in macrophage-derived EVs might represent a secretion pathway to tissue calcification sites and/or a modulating role on the calcification potential of macrophage-derived EVs. Additionally, we found mRNA for GRP and MGP on the EVs isolated from macrophages. EVs, particularly exosomes, are known to carry genetic material such as mRNA and miRNA, acting as genetic transfer vehicles and possibly modulating gene expression of target cells [53–55]. These findings open new perspectives on the role of GRP in chronic inflammation and calcification-related pathologies, and should also be highly relevant to our current molecular knowledge on intercellular communication involved in physiological and pathological processes, warranting further investigation.

It should be noted that although THP-1 immortalized cell line might not entirely reflect the biological function of monocytes/macrophages, our previously published studies showing the anti-inflammatory potential of GRP in articular cells [16], its anti-mineralization function, and its presence in immune cells associated to different pathologies [16, 31], strongly support the findings and conclusions presented in this work. Nevertheless, the anti-inflammatory function of GRP should be further corroborated using primary monocytes and macrophage cells, and the association of GRP with pathological conditions such as atherosclerosis should be evaluated *in vivo* focusing on inflammatory events. Although we have focused this work on the role of GRP in inflammatory processes, the detection of MGP in inflammatory cells and its differential expression pattern during inflammation in THP-1 and THP-1 MoM cells, foresee a possible involvement of MGP in inflammation that should be further addressed in the future.

### Conclusions

Overall, the novel data presented here indicate a strong association of GRP and the immune system, acting as an anti-inflammatory agent in monocyte and macrophage cells. We propose that in a context of chronic inflammation and calcification-related pathologies, GRP might act as a molecular mediator in the inflammation and calcification paradigm, emphasizing its potential for future therapeutic application.
