# Correction: Gla-rich protein function as an anti-inflammatory agent in monocytes/macrophages: Implications for calcification-related chronic inflammatory diseases

**DOI:** 10.1371/journal.pone.0192875

**Published:** 2018-02-08

**Authors:** The PLOS ONE staff

The following information is missing from the Competing Interests section: The tools and methods described in this manuscript are included in a PCT patent application PCT/PT2009000046, which is owned by University of Algarve and the Center of Marine Sciences (CCMAR). Dina C. Simes and Carla S.B. Viegas are cofounders of GenoGla Diagnostics; Cees Vermeer is founder of VitaK. This does not affect our adherence to PLOS's policies on sharing data and materials.
